# Complete remission of advanced hepatocellular carcinoma following transient chemoembolization and portal vein ligation

**DOI:** 10.1186/s40792-018-0510-8

**Published:** 2018-08-29

**Authors:** Yuki Koga, Toru Beppu, Katsunori Imai, Kunitaka Kuramoto, Tatsunori Miyata, Yuki Kitano, Shigeki Nakagawa, Hirohisa Okabe, Kazutoshi Okabe, Yo-ichi Yamashita, Akira Chikamoto, Hideo Baba

**Affiliations:** 1 Department of Surgery, Yamaga City Medical Center, Kumamoto, Japan; 2 Department of Gastroenterological Surgery, Graduate School of Life Sciences Kumamoto University, 1-1-1 Honjo, Chuo-ku, Kumamoto, 860-8556 Japan

**Keywords:** Hepatocellular carcinoma, Portal vein tumor thrombus, Peritoneal dissemination, Complete remission, Chemoembolization, Portal vein ligation

## Abstract

**Background:**

Macroscopic diffuse-type hepatocellular carcinoma with concomitant major portal vein tumor thrombus (PVTT) and peritoneal dissemination indicates poor prognosis. Additionally, triple-positive tumor marker status is a predictor of poor outcome even after hepatectomy. Sorafenib is recommended in such patients, but it has limited therapeutic effectiveness.

**Case presentation:**

A 54-year-old man was diagnosed with a liver abscess that was treated by puncture and drainage at a regional hospital. However, the diagnosis was subsequently changed to hepatocellular carcinoma with macroscopic portal vein tumor thrombus, based on the results obtained for the triple-positive tumor markers (alpha-fetoprotein, 45,928 ng/ml; protein induced by vitamin K absence or antagonist-II, 125,350 mAU/ml; and alpha-fetoprotein-L3, 38.3%). As the patient’s liver functional reserve was not adequate for curative resection, chemoembolization was performed with a hepatic arterial infusion of cisplatin (50 mg) and 5-FU (1000 mg), followed by mild embolization with cisplatin (50 mg) suspended in lipiodol (5 ml) and starch microspheres (300 mg) containing mitomycin C (4 mg). As the thrombus had progressed to the bifurcation of the right and left portal veins, the right vein was surgically ligated. Three peritoneal nodules could be identified and were removed. Three additional rounds of hepatic arterial chemotherapy/chemoembolization were performed after the initial surgery. At the 2-year evaluation, all tumor markers were observed to have normalized and diagnostic imaging showed complete remission.

**Conclusions:**

Complete remission of hepatocellular carcinoma with macroscopic portal vein tumor thrombus and peritoneal dissemination was obtained with a treatment regimen that involved four rounds of hepatic arterial infusion chemotherapy and transient chemoembolization, portal vein ligation, and the removal of peritoneal dissemination. This regimen can be recommended for patients with advanced hemiliver lesions who cannot undergo curative resection.

## Background

Macroscopic diffuse-type hepatocellular carcinoma (HCC) with concomitant major portal vein tumor thrombus (PVTT) and peritoneal dissemination suggests poor prognosis in the patients with HCC [1–4]. A triple-positive tumor marker status also predicts a poor outcome [5–7], and even though patients with advanced HCC are often treated with sorafenib, tumor control and survival rates remain unsatisfactory [8, 9]. Chemoembolization is an option for treating advanced HCC; however, it is contraindicated in patients with a main to first portal vein branch [10]. In such patients, hepatic arterial infusion chemotherapy (HAIC), followed by transient chemoembolization using temporary embolic materials, is an alternative treatment option [11, 12]. Portal vein embolization (PVE) or portal vein ligation (PVL) can broaden the indications for liver resection in patients with HCC and major PVTT [13]. Even in unresectable HCC with macroscopic PVTT, PVE can avoid metastasis of the non-embolized liver and can improve overall survival rate [14]. We report a case of complete remission of a diffuse-type HCC with PVTT after four rounds of transient chemoembolization combined with surgical PVL and extirpation of peritoneal dissemination.

## Case presentation

**Fig. 1:**
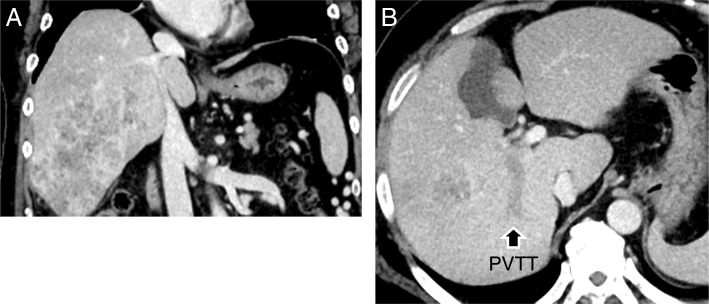
Dynamic CT scan on admission to our hospital. **a** Coronal view (portal phase). **b** Axial view (portal phase). Dynamic CT showed a large diffuse-type HCC with a PVTT in the right posterior branch of the portal vein (arrow)

A 54-year-old man with a history of diabetes mellitus and hypertension was admitted to a regional hospital because of high fever and right hypochondriac pain. Hepatitis B virus surface antigen and hepatitis C virus antibody were both found to be negative, but he showed evidence of an excessive inflammatory reaction. A diagnosis of liver abscess was carried out that was managed by immediately performing a percutaneous puncture with drainage. Laboratory evaluation (Table 1) found poor liver function and very high levels of alpha-fetoprotein (AFP, 45,928 ng/ml; normal, ≤ 20 ng/ml), protein induced by vitamin K absence or antagonist-II (PIVKA-II, 125,350 mAU/ml; normal, ≤ 40 mAU/ml), and AFP-L3 (38.3%, normal, ≤ 10%). The patient was diagnosed with HCC and with the triple-positive tumor marker status indicating highly malignant disease [5, 6]. The patient was also found to have a portal vein tumor thrombosis in the right posterior branch of the portal vein (Fig. 1). Although a right hepatectomy was indicated for curative resection, residual liver function of the remnant volume was estimated to be insufficient [15, 16].

**Table 1:** Laboratory values on admission

T-protein	7.2	g/dl	AFP	45,928	ng/ml
Albumin	1.9	g/dl	PIVKA-II	125,350	AU/ml
T-bilirubin	1.2	mg/dl	AFP-L3	38.3	%
D-bilirubin	0.6	mg/dl			
ALT	30	U/L	HBs-Ag	(−)	
AST	136	U/L	HBs-Ab	(−)	
LDH	468	U/L	HBc-Ab	(−)	
ALP	992	U/L	HCV-Ab	(−)	
γ-GTP	524	U/L			
Cholinesterase	79	U/L	White blood cell	12.38	× 10^3^/μL
			Neutrophils	86.2	%
			Red blood cell	3.55	× 10^6^/μL
BUN	10.1	mg/dl	Hemoglobin	10.1	g/dl
Creatinine	0.41	mg/dl	Platelet	343	× 10^3^/μL
FBS	106	mg/dl	CRP	25.01	mg/dl
Hb A1c	6.3	%	PT activity	54.2	%
			ICG R15	32.6	%

*ALT* alanine transaminase, *AST* aspartate aminotransferase, γ-GTP, γ-glutamyl transpeptidase, *LDH* lactate dehydrogenase, *ALP* alkaline phosphatase, *BUN* blood urea nitrogen, *FBS* fasting blood glucose, *Hb* hemoglobin, *AFP* alpha-fetoprotein, *PIVKA-II* protein induced by vitamin K absence or antagonist-II, *HBs-Ag and HBs-Ab* hepatitis B virus surface antigen and antibody, *HBc-Ab* hepatitis B virus core antibody, *HCV-Ab* hepatitis C virus antibody, *CRP* C-reactive protein, *PT* prothrombin time, *ICGR15* indocyanine green retention rate at 15 min

The patient was initially treated with chemoembolization (Table 2) using a HAIC of cisplatin (50 mg/100 ml/10 min) and 5-FU (1000 mg/100 ml/10 min), followed by cisplatin (50 mg) suspended in lipiodol (5 ml) and starch microspheres (300 mg) containing mitomycin C (4 mg) [11, 12]. After the first round of chemoembolization, examination showed incomplete lipiodol accumulation within the tumor. Additionally, as the PVTT progressed to the right main portal vein, surgical PVL was performed to avoid involvement of the left portal vein. Three disseminated peritoneally nodules were also removed. Three additional rounds of transient chemoembolization were performed after the initial surgical procedure.

**Table 2:** Hepatic arterial infusion and chemoembolization treatment regimen

	First	Second	Third	Fourth
Cisplatin solution	50 mg	50 mg	50 mg	80 mg
5-FU solution	1000 mg	1000 mg	1000 mg	1000 mg
Cisplatin/lipiodol suspension	50 mg/5.0 ml	45 mg/4.5 ml	30 mg/3.0 ml	–
Farmorubicin/lipiodol emulsion	–	–	–	20 mg/2.0 ml
MMC/Spherex	4 mg/300 mg	4 mg/300 mg	4 mg/300 mg	4 mg/180 mg

*5-FU* 5-fluorouracil, *MMC* mytomycin C

**Fig. 2:**
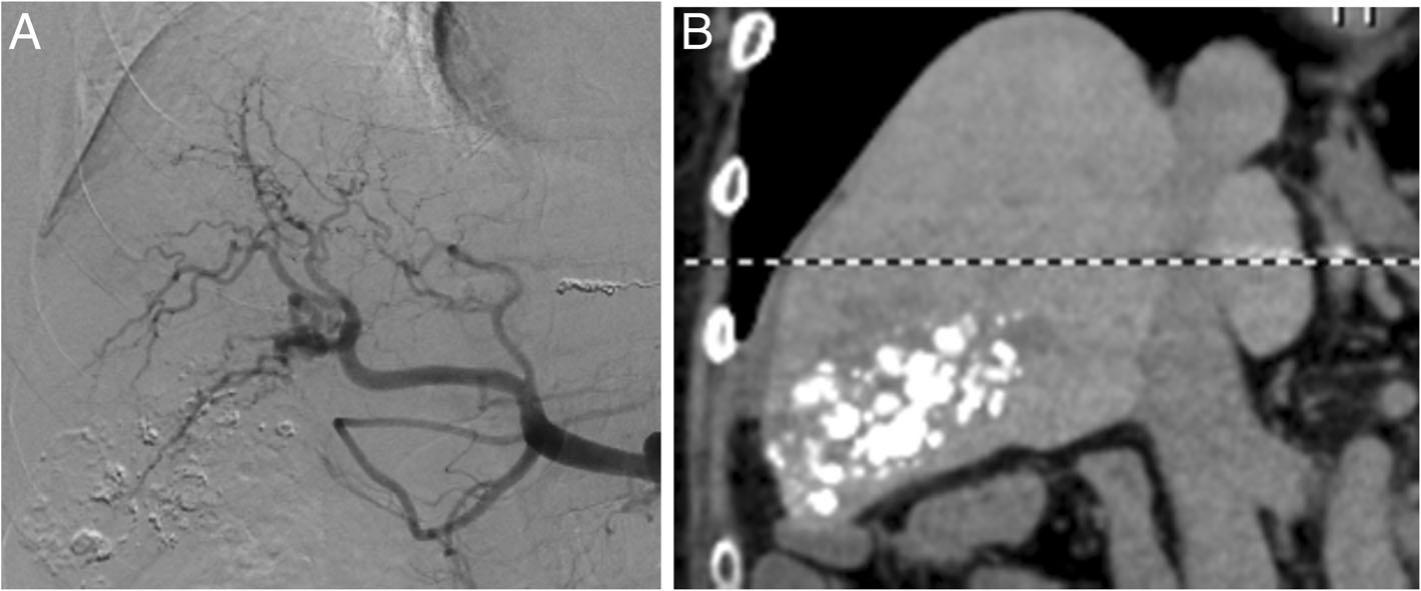
Diagnostic images at the fourth chemoembolization procedure. **a** Digital subtraction angiography. **b** Plain CT after chemoembolization. The main tumor is markedly diminished with no enhancement, and lipiodol showed spotty but strong accumulation

**Fig. 3:**
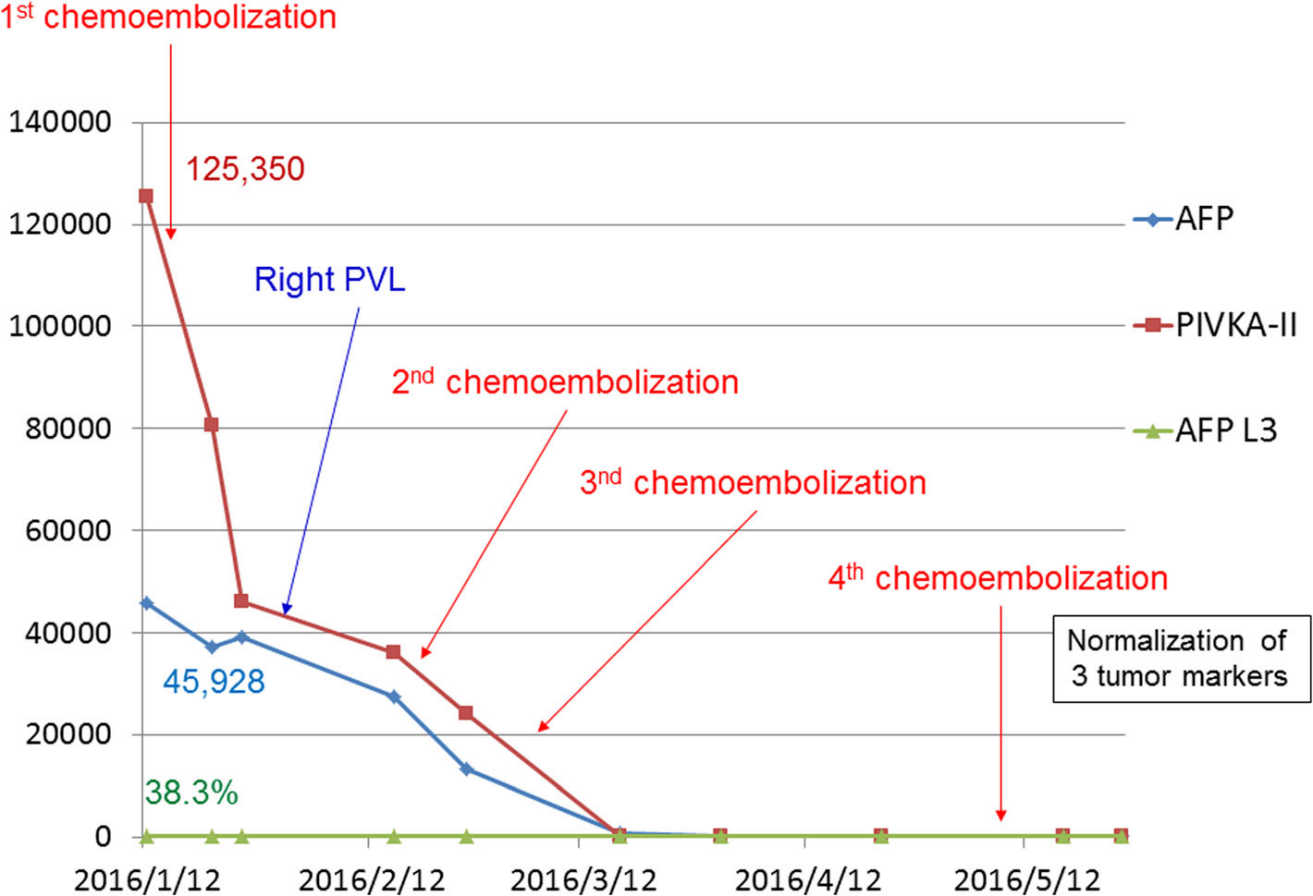
Treatment course and changes in tumor markers. Tumor markers were abnormally high before the first chemoembolization, but they remained within the normal range for 18 months after the fourth chemoembolization procedure

At the time of the fourth chemoembolization, the tumors responded to the treatment and markedly reduced in size without enhancement (Fig. 2). Further, no new tumors were found in the liver, and the tumor markers returned to their normal levels (Fig. 3). A suspicious lesion (2 cm in diameter) recurred at15 months after the initial treatment, which was treated with percutaneous radiofrequency ablation. The patient is alive at 2-year post-procedure and shows complete remission, as defined by the modified response evaluation criteria in solid tumor criteria.

### Discussion

This patient achieved complete remission after chemoembolization, surgical PVL, and extirpation of peritoneally disseminated nodules. The case was complicated by the poor prognostic factors, including the macroscopic diffuse-type classification, a macroscopic PVTT, the peritoneal dissemination, and triple-positive tumor marker status [1–6]. A tumor biopsy was not performed, but the presence of a poorly differentiated HCC was strongly suggested by the tumor marker status and diagnostic imaging [5–7].

In patients with HCC and macroscopic PVTT, multidisciplinary treatment, including liver resection, provides an excellent prognosis [17]. Moreover, a recent nationwide survey in Japan indicated that liver resection was more effective than non-surgical treatment in cases with a PVTT that is limited to the first- or second-order branches [18]. Multiple measurements of the liver function and functional liver volume after PVL [15, 16, 19] in our patient indicated that liver resection was not a viable option. For such HCC patients, other treatment options such as HAIC with chemoembolization and sorafenib also result in poor median survival times of 3.5–10.2 and 8.1–8.9 months, respectively [17, 20]. However, right portal vein occlusion can prevent both progression of the right PVTT into the left or main portal vein and intrahepatic metastasis into the left liver [13, 14, 21], and it may also enhance the effectiveness of HAIC because capsular invasion and satellite nodules could be supplied by the portal vein with hepatic artery [14, 22]. While formulating the treatment strategy, we also considered the fact that PVE is not indicated in patients with a PVTT that is in close proximity to the bifurcation.

Peritoneal dissemination of HCC can occur after tumor rupture or due to therapeutic interventions. The standard treatment for dissemination of HCC would be systemic chemotherapy, and if dissemination is localized to abdominal cavity or abdominal wall, then the surgical removal for dissemination of HCC might be a challenging option [23, 24]. In this patient, iatrogenic seeding may have occurred by tumor puncture when drainage was started. However, the spread was limited, and all lesions could be isolated and surgically removed.

Our patient was treated by HAIC followed by transient chemoembolization. Cisplatin and 5-FU are effective for HCC, evidently in intra-arterial infusion [25, 26]. In fact, some patients with advanced HCC and PVTT have reportedly shown complete clinical remission or pathological response after this regimen [27–29]. Cisplatin modulates 5-FU activity, and the two drugs seem to have a synergistic effect. Further, as cisplatin infused via the hepatic artery is not trapped in the liver parenchyma, it would also be effective as systemic chemotherapy. Essentially, cisplatin suspended in lipiodol is a highly effective embolic material that is also used in HCC treatment [26, 30, 31]. Mitomycin-C and degradable starch microspheres provide temporary occlusion, which may also increase drug concentration [11].

Sorafenib is effective in HCC patients with macroscopic vascular invasions, extrahepatic spread, or both, but a recent trial has reported a response rate of 2% and a median survival time of only 10.7 months [8]. However, a few cases of complete remission after sorafenib therapy have been reported [32, 33]. In our patient, dynamic imaging detected no viable HCC and persisting normalization of the three tumor markers. Previous reports suggest that HAIC with a cisplatin–lipiodol suspension combined with 5-FU can lead to better response rates and overall survival rates (without extrahepatic metastasis) compared to only sorafenib in patients with advanced HCC and PVTT [26]. Thus, it would be possible to administer additional chemoembolization or radiofrequency ablation for intrahepatic recurrence and sorafenib therapy for extrahepatic metastasis. It has similarly been reported that sorafenib is effective in patients with HCC refractory to chemoembolization [34] and that sorafenib and HAIC with cisplatin may have synergistic effects [35].

The maintenance of liver function is the key to achieving longer survival in advanced HCC patients, and it is known that effective treatment for advanced HCC can improve liver function [36]. Further, it has been reported that a Child–Pugh score of ≤ 7 shows a better response to HAIC with better prognosis compared with Child–Pugh score of 8 or 9 [37]. However, our patient had a Child–Pugh score of 8 at admission, which improved to 6 after multidisciplinary treatment, indicating that the treatment regimen was effective.

## Conclusions

A treatment consisting of chemoembolization and surgical intervention, including PVL, may allow complete remission in patients with advanced hemiliver lesions, PVTT, and/or localized peritoneal dissemination.

## Abbreviations

AFP: Alpha-fetoprotein
ALP: Alkaline phosphatase
ALT: Alanine transaminase
AST: Aspartate aminotransferase
BUN: Blood urea nitrogen
CRP: C-reactive protein
CT: Computed tomography
FBS: Fasting blood glucose
Hb: Hemoglobin
HBc-Ab: Hepatitis B virus core antibody
HBs-Ag and HBs-Ab: Hepatitis B virus surface antigen and antibody
HCC: Hepatocellular carcinoma
HCV-Ab: Hepatitis C virus antibody
γ-GTP: γ-Glutamyl transpeptidase
ICGR15: Indocyanine green retention rate at 15 min
LDH: Lactate dehydrogenase
PIVKA-II: Protein induced by vitamin K absence or antagonist-II
PT: Prothrombin time
PVTT: Portal vein tumor thrombus
PVL: Portal vein ligation
PVE: Portal vein embolization
RECIST: Response evaluation criteria in solid tumors
